# Hospital admissions of acute cerebrovascular diseases during and after the first wave of the COVID-19 pandemic: a state-wide experience from Austria

**DOI:** 10.1007/s00415-021-10488-8

**Published:** 2021-02-27

**Authors:** Thomas Gattringer, Simon Fandler-Höfler, Markus Kneihsl, Edith Hofer, Wolfgang Köle, Reinhold Schmidt, Karl-Heinz Tscheliessnigg, Almut-Michaela Frank, Christian Enzinger

**Affiliations:** 1 Department of Neurology, Medical University of Graz, Auenbruggerplatz 22, 8042 Graz, Austria; 2 Division of Neuroradiology, Vascular and Interventional Radiology, Department of Radiology, Medical University of Graz, Graz, Austria; 3 Institute for Medical Informatics, Statistics and Documentation, Medical University of Graz, Graz, Austria; 4 Medical Directorate, University Hospital Graz, Graz, Austria; 5 Steiermärkische Krankenanstaltengesellschaft m.b.h, Graz, Austria

**Keywords:** Ischaemic stroke, Intracranial haemorrhage, Transient ischaemic attack, Acute stroke care, Corona virus disease 2019

## Abstract

We investigated hospital admission rates for the entire spectrum of acute cerebrovascular diseases and of recanalization treatments for ischaemic stroke (IS) in the Austrian federal state of Styria during and also after the first coronavirus disease 2019 (COVID-19) wave. We retrospectively identified all patients with transient ischaemic attack (TIA), IS and non-traumatic intracranial haemorrhage (ICH; including intracerebral, subdural and subarachnoid bleeding types) admitted to one of the 11 public hospitals in Styria (covering > 95% of inhospital cerebrovascular events in this region). Information was extracted from the electronic medical documentation network connecting all public Styrian hospitals. We analysed two periods of interest: (1) three peak months of the first COVID-19 wave (March–May 2020), and (2) three recovery months thereafter (June–August 2020), compared to respective periods 4 years prior (2016–2019) using Poisson regression. In the three peak months of the first COVID-19 wave, there was an overall decline in hospital admissions for acute cerebrovascular diseases (RR = 0.83, 95% CI 0.78–0.89, *p* < 0.001), which was significant for TIA (RR = 0.61, 95% CI 0.52–0.72, *p* < 0.001) and ICH (0.78, 95% CI 0.67–0.91, *p* = 0.02), but not for IS (RR = 0.93, 95% CI 0.85–1, *p* = 0.08). Thrombolysis and thrombectomy numbers were not different compared to respective months 4 years prior. In the recovery period after the first COVID-19 wave, TIA (RR = 0.82, 95% CI 0.71–0.96, *p* = 0.011) and ICH (RR = 0.86, 95% CI 0.74–0.99, *p* = 0.045) hospitalizations remained lower, while the frequency of IS and recanalization treatments was unchanged. In this state-wide analysis covering all types of acute cerebrovascular diseases, hospital admissions for TIA and ICH were reduced during and also after the first wave of the COVID-19 pandemic, but hospitalizations and recanalization treatments for IS were not affected in these two periods.

## Introduction

The outbreak of the coronavirus disease 2019 (COVID-19) pandemic led to a reorganisation of the health care system and had a global impact on acute medical care for other major diseases. Data from different regions indicate that stroke care was particularly affected with limited pre- and intrahospital care, and consecutive drops in hospital admissions and acute recanalization treatments. [1–3] However, most reports derive from countries/regions that were severely affected by the viral pandemic (potentially entailing some publication bias) and mainly concentrated on ischaemic stroke (IS). [4–8] Moreover, information on hospital admissions in the recovery period after the first COVID-19 wave is lacking. Therefore, we analysed the influence of the COVID-19 outbreak on hospitalisation rates during and after the first wave of the pandemic for the entire spectrum of acute cerebrovascular diseases including transient ischemic attack (TIA), IS and intracranial haemorrhage (ICH) including all non-traumatic bleeding types in the Austrian federal state of Styria; a region with 1.24 million inhabitants that was only modestly affected by the first COVID-19 wave.

## Methods

We retrospectively identified all patients with TIA, IS and non-traumatic ICH (intracerebral, subdural and subarachnoid bleeding types) according to ICD-10 diagnoses admitted to one of the 11 public hospitals in Styria, covering > 95% of all acute cerebrovascular diseases treated in a hospital in this region (data derived from the Styrian health care fund). Information was extracted from the fully electronic medical documentation network connecting all Styrian public hospitals. In our region, specialised stroke care is provided by four regional neurological departments (including dedicated stroke units) and one comprehensive stroke centre additionally offering endovascular therapy and neurosurgical care (University Hospital of Graz). In Styria, TIA patients are almost exclusively admitted to the hospital and treated at neurological departments.

In Austria, first COVID-19 cases were recognised on February 25, 2020 with a rapid increase at the beginning of March. Restrictive social measures started on March 16, a stepwise opening of social lockdown was deployed until end of May. [10] Therefore, our analysis covered two time periods of interest: (1) three peak months of the first COVID-19 wave (March–May), and (2) three recovery months thereafter (June–August) compared to respective periods 4 years prior (2016–2019). Notably, in Austria and particularly in Styria, neurological emergency services including outpatient clinics were maintained during the COVID-19 crisis.

We used Poisson regression to compare number of cases and present rate ratios (RR) with 95% confidence intervals (CI).

Statistical analysis was performed using IBM SPSS Statistics 26 with a *p* value of < 0.05 considered significant. Investigators did not have access to identifiable protected health information, thus, no ethics committee vote was required.

## Results

An overview of hospital admissions for COVID-19 and acute cerebrovascular diseases as well as thrombolysis and thrombectomy numbers for January–August 2020 is presented in Table 1.

**Table 1:** Hospital admissions for patients with COVID-19 and acute cerebrovascular events as well as numbers of thrombolysis and thrombectomy in Styria from January to August 2020

	Months January–August 2020							
	Jan	Feb	Mar	Apr	May	Jun	Jul	Aug
COVID-19 new infections, *n*	0	0	1087	639	73	42	228	435
Median COVID-19 infections *(min–max)*	0	0	366 (18–1016)	925 (541–1129)	279 (190–529)	178 (151–194)	249 (190–272)	265 (235–417)
Hospitalised COVID-19 patients *(n)*	0	0	94	298	65	21	21	28
Acute cerebrovascular diseases *(n)*	431	352	380	298	336	339	390	350
Transient ischaemic attack *(n)*	77	71	49	48	62	69	74	63
Ischemic stroke *(n)*	257	202	262	193	212	199	240	229
Intravenous thrombolysis *(n)* (% ischemic stroke patients)	46 18%	34 17%	44 17%	29 15%	37 17%	35 18%	36 15%	34 15%
Mechanical thrombectomy *(n)* (% ischemic stroke patients)	10 4%	12 6%	14 5%	7 4%	9 4%	7 4%	8 3%	11 5%
Intracranial haemorrhage, total *(n)*	97	79	69	57	62	71	76	58
Intracerebral *(n)*	39	27	29	23	22	21	24	22
Subdural *(n)*	33	29	21	21	14	31	31	23
Subarachnoid *(n)*	25	22	19	13	26	19	21	13

**Fig. 1:**
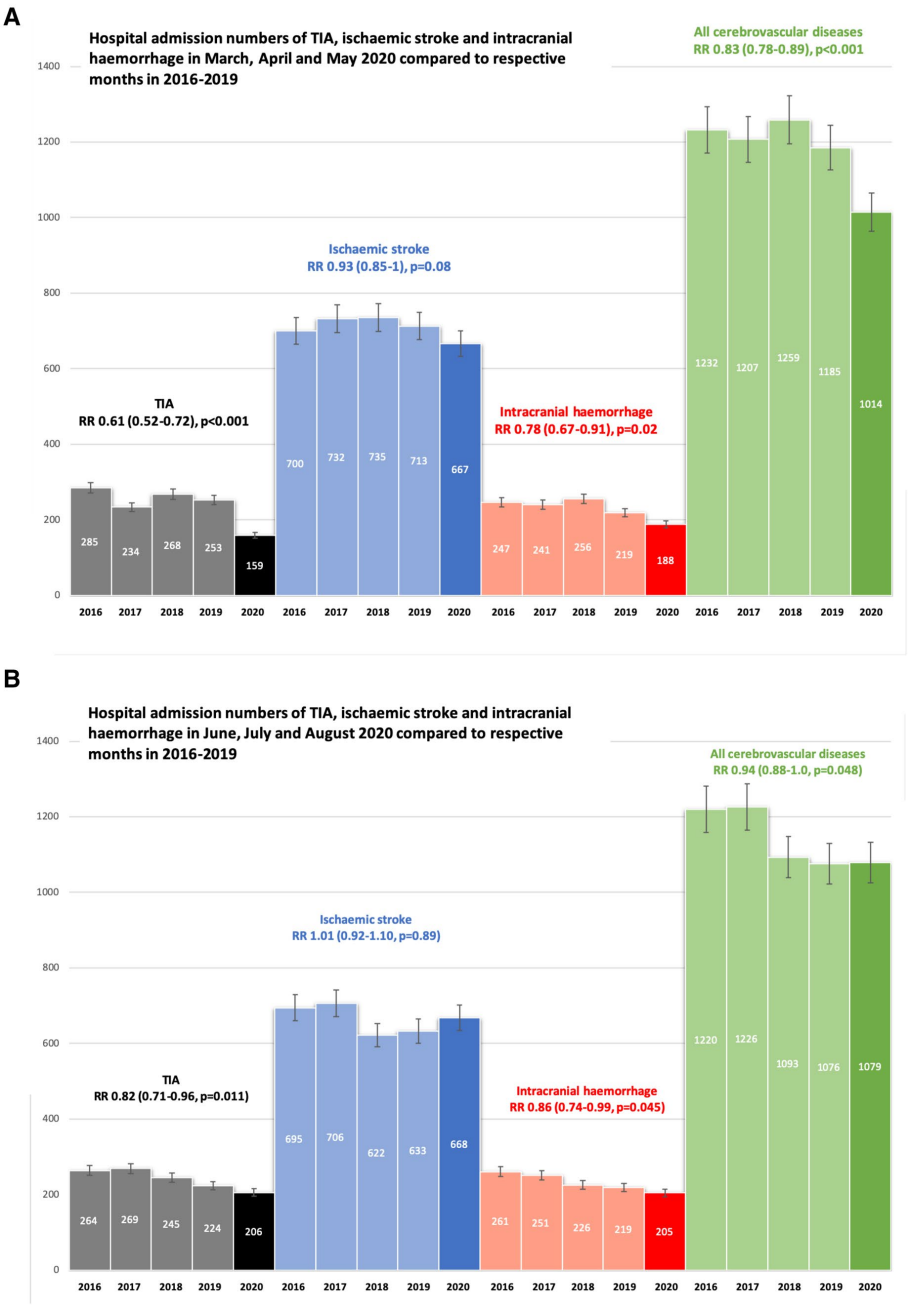
Hospitalisation for cerebrovascular diseases in Styria in March, April and May (first COVID-19 wave, panel **a**) and June, July and August (recovery period, panel **b**) 2020 compared to respective months in 2016–2019

In March–May 2020, there was a significant decline in hospital admissions for TIA, but not for IS (Fig. 1a). Thrombolysis (RR 1.20, 95% CI 0.97–1.49, *p* = 0.09) and thrombectomy (RR 1.19, 95% CI 0.79–1.80, *p* = 0.41) numbers did also not change during the peak months of the first COVID-19 wave. However, ICH hospitalizations significantly decreased (Fig. 1a). Regarding ICH subtypes, diminished rates were observed for intracerebral (RR 0.73, 95% CI 0.57–0.94, *p* = 0.015) and subdural (RR 0.68, 95% CI 0.51–0.90, *p* = 0.006), but not for subarachnoid haemorrhage (RR 1.02, 95% CI 0.76–1.36, *p* = 0.91). The rate of all acute cerebrovascular diseases combined was reduced (RR 0.83, 95% CI 0.78–0.89, *p* < 0.001) (Fig. 1a).

Figure 1B illustrates hospitalizations for acute cerebrovascular diseases in the 3 months after the first COVID-19 wave compared to 2016–2019. Admission for TIA, ICH and total cerebrovascular diseases remained lower, while the frequency of IS was unchanged. Furthermore, thrombolysis (RR 1.20, 95% CI 0.95–1.47, *p* = 0.13) and thrombectomy (RR 1.27, 95% CI 0.82–1.97, *p* = 0.29) numbers remained constant.

## Discussion

In line with studies from other regions, we found overall reduced hospitalizations for acute cerebrovascular diseases during the first COVID-19 wave [2–9]. While this was significant for TIA and ICH, IS admissions as well as thrombolysis and thrombectomy numbers were unchanged compared to previous years. It is plausible that fewer patients with transient/minor stroke symptoms sought medical attention at the height of the pandemic, possibly because of fear contracting the virus or reduced health care capacities. However, hospital access should not have been a major problem in our region as the COVID-19 burden was rather low in Styria in the analysed periods, and IS care was not affected.

Notably, these assumptions would not explain reduced ICH rates seen in our region, as ICH is the most severe stroke subtype and it is doubtful that patients with ICH would stay at home, especially in our well-developed health care system. Rather, it is conceivable that the COVID-19 pandemic led to an actual decrease in ICH, potentially related to reduced levels of stress, air pollution, fast food (sodium) consumption and most notably blood pressure crisis—the most important trigger factor for ICH. [11]

Nevertheless, it needs to be noted that there is an indication for a trend towards declining rates of hospitalizations for the different events assessed (considering fluctuations over years), which also affects ICH. However, a similar drop in ICH admissions during the COVID-19 crisis was also found in other cohorts. [8, 12, 13]

To our knowledge, this is one first systematic report on admissions for acute cerebrovascular diseases investigating the recovery period after the first COVID-19 wave. Interestingly, hospitalizations remained lower compared to 2016–2019, being significant for TIA and ICH, and there was also no increase in IS. Such excess could have been expected as a potential complication of less intensive care for TIA patients during the COVID-19 peak months. Our results argue for a real population-level-reduction of cerebrovascular events related to the viral pandemic, which needs to be studied further prospectively on a long-term basis. Another strength of our work is the analysis of the entire spectrum of acute cerebrovascular diseases including TIA, IS and different types of ICH in a clearly defined state-wide system of acute medical care.

Limitations of our work include reliance on ICD-10 coding with the possibility of some misclassification and missing data on stroke severity, aetiology and outcome, but this was beyond the scope of our work. Despite that, this region-wide analysis adds novel information on hospitalizations for the entire spectrum of cerebrovascular diseases during and after the first COVID-19 wave. These findings are relevant for policy makers and healthcare planners for subsequent COVID-19 waves or new (viral) pandemics.

## Data Availability

The data that support the findings of this study are available from the corresponding author upon reasonable request.
